# In This Issue

**DOI:** 10.1002/1097-0061(200012)17:4<::AID-YEA50>3.0.CO;2-U

**Published:** 2000


**Yeast**
*Yeast* 2000; **17**: v–vi.

### In this Issue

### Comparison of *Mycobacterium tuberculosis* isolates reveals deletion as an important factor in variation

Ho *et al.* have made a study of a 20 kb variable region in 24 different clinical isolates of *M. tuberculosis*. The strains contained either genomic deletions or insertions of one or more copies of the *IS6110* insertion element in this region. It appears that recombination between adjacent elements could be the cause of the deletions. This could then be an important mechanism for variation in *M. tuberculosis*, which shows remarkably low polymorphism of structural genes.

### A new computational approach to assigning functional categories, applied to *Escherichia coli* and *M. tuberculosis*

Annotation is certainly the slow step of all sequencing projects. One part of this is the use of bioinformatic approaches to assign putative functional categories to genes. Automation of this step would make a significant reduction in the time taken to analyse genomics data. In their paper, King *et al.* describe an inductive logic programming (ILP) approach that uses clustering and rule learning. They have tested this approach to categorizing genes on *M. tuberculosis* and *E. coli*, achieving predicted functional categories for 65% and 24%, respectively, of those genes with no assigned function.

### Morpholino knock-out of VEGF-A in zebrafish reveals requirements for angiogenesis

Nasevicius *et al.* have used morpholino oligos, directed towards the VEGF-A gene, to block its function in zebrafish. A demonstration of the application of this technique to the zebrafish has been eagerly awaited by the community. This work is also a crucial breakthrough in the study of angiogenesis, because it has not previously been possible to study the function of VEGF-A, since mouse knock-outs show dominant haplo-insufficiency. The resulting 'morphant' fish show that there are different requirements for the specification of axial and intersegmental vascular structure.

### Morphants: a new systematic vertebrate functional genomics approach

In his review, Steve Ekker describes the various sequence-dependent approaches that have been tried (with little success so far) for the selective knock-out of gene expression in zebrafish. The story concludes with a detailed explanation of experimental approaches for, and limitations of, the use of morpholino oligos. This technique has now been successfully used in zebrafish (see Nasevicius *et al.*, this issue), to explore the role of VEGF-A angiogenesis.

### Plant pathogen sequencing

The recent completion of the sequencing of *Xylella fastidiosa* by a consortium of laboratories in the state of Sao Paolo, Brazil was a landmark event, being the first time that a plant pathogen genome has been sequenced in its entirety. Several *Xylella* genes show strong homology to *X. campestris* virulence genes or xanthan gum biosynthesis genes. Mike Daniels and Max Dow highlight the similarities and differences between the organization and the products of these genes, which have implications for the study of *X. fastidiosa* pathogenesis. The completion of the *Xylella* sequence turns thoughts to functional genomics of plant pathogens, and the review concludes with a discussion of the future prospects for functional genomics studies directed at identifying pathogenicity genes of *Xylella* and related phytopathogens.

### Featured Organism: pathogen special

Microbial genomes are now being published at an impressive rate. In this issue, we feature the three

Copyright © 2000 John Wiley & Sons, Ltd.

most recent arrivals, giving you a guide to *Xylella fastidiosa*, *Vibrio cholerae* and *Pseudomonas aeruginosa*. *X. fastidiosa* is the cause of citrus variegated chlorosis (CVC), in which hard, juiceless fruits are produced, due to blockage of the xylem by the bacteria. *V. cholerae* is the causative agent of cholera, colonizing the small intestine of humans, causing severe, watery diarrhoea, which often leads to death. *P. aeruginosa* is an opportunistic pathogen that can affect those with compromised immune systems, such as cystic fibrosis sufferers and patients on respirators. It causes progressive damage to the lungs, eventually leading to death.

### Meeting Highlights: Beyond the Genome 2000, IUBMB and FEBS joint meeting

From comparative genomics and evolutionary studies to new approaches for functional genomics, we bring you a report detailing the most exciting presentations from this joint international meeting.

### Meeting Review: American Society for Mass Spectrometry

Francesco Brancia reports on the most relevant talks and posters that were presented at this crucial meeting for the community. The take-home message appears to be that the application of mass spectrometry to proteomics is a rapidly growing field, which is spawning technological advances, and everyone wants to be part of it.

### Website Review: InterPro

Staying with the proteomics theme, Chris Southan reviews the newest protein database provided by EMBL, InterPro. This contains their highest quality data, with detailed annotation, provided as a result of their integration of several family and motif recognition systems: PROSITE, PRINTS, Pfam and ProDom. Taking HtrA/DegQ proteases as examples, he demonstrates the strengths of this new resource for comparative genomics.

### Website Update: UK CropNet

Things can move quickly on the Internet. Sometimes the top sites update their information and add new tools faster than we get to grips with the existing ones. Keith Bradnam and Sean May describe the new additions to the UKCropNet site since our review in the last issue of *CFG*.

Copyright © 2000 John Wiley & Sons, Ltd.

